# Author Correction: Investigating the presence of adsorbed species on Pt steps at low potentials

**DOI:** 10.1038/s41467-022-31404-2

**Published:** 2022-06-28

**Authors:** Rubén Rizo, Julia Fernández-Vidal, Laurence J. Hardwick, Gary A. Attard, Francisco J. Vidal-Iglesias, Victor Climent, Enrique Herrero, Juan M. Feliu

**Affiliations:** 1 Instituto de Electroquímica, Universidad de Alicante, Apdo. 99, E-03080 Alicante, Spain; 2 Stephenson Institute for Renewable Energy, University of Liverpool, Peach Street, Liverpool, L69 7ZF UK; 3 Department of Physics, University of Liverpool, Crown Street, Liverpool, L69 7ZD UK

**Keywords:** Electrocatalysis, Electrocatalysis, Electrocatalysis, Catalytic mechanisms

Correction to: *Nature Communications* 10.1038/s41467-022-30241-7, published online 10 May 2022.

The original version of this Article omitted references to previous work when describing the state of the art of the subject. This has been added as reference [24,25,26] at: ‘It has been generally assumed that the hydrogen adsorption/desorption process is responsible for this peak, although some results suggest that OH adsorption can also be involved in these processes^22-24^. Indeed, DFT results suggest that cation coadsorption with OH is responsible for the observed voltammetric behaviour^25,26^.’ This has been corrected in the PDF and HTML versions of the Article.

