# Supplementary material for: Investigating the presence of adsorbed species on Pt steps at low potentials
Source: Nat Commun. 2022 May 10;13:2550. doi: 10.1038/s41467-022-30241-7 (PMC9090771; doi:10.1038/s41467-022-30241-7)
Supplement: Supplementary file 1 — Supplementary Information [file 41467_2022_30241_MOESM1_ESM.pdf]

SUPPORTING INFORMARTION

Investigating the presence of adsorbed species on Pt steps at low potentials

Rubén Rizo[1*], Julia Fernández-Vidal[2], Laurence J. Hardwick[2,], Gary A. Attard[3], Francisco J. Vidal-Iglesias[1], Victor Climent[1], Enrique Herrero[1]*, Juan M. Feliu[1]*

[1]*Instituto de Electroquímica, Universidad de Alicante,*

Apdo. 99, E-03080, Alicante, Spain.

[2]*Stephenson Institute for Renewable Energy, Department of Chemistry, University of Liverpool, Peach Street, Liverpool L69 7ZF, United Kingdom*

[3]*Department of Physics, University of Liverpool, Crown Street, Liverpool, L69 7ZD, United Kingdom*

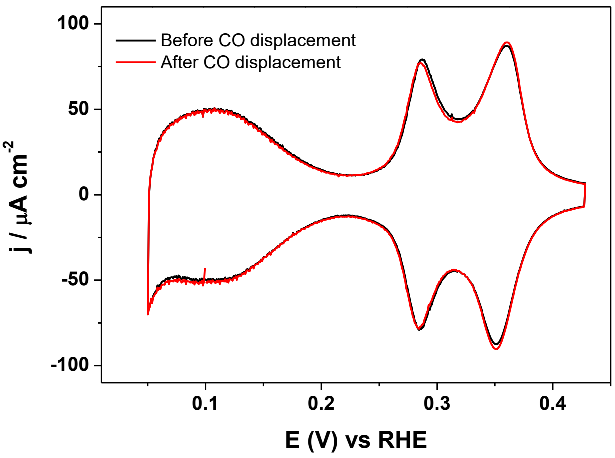

Figure S1. Cyclic voltammetry for Pt(311) in 0.1 M $HClO_4$ solution before and after the CO displacement experiments.

Note 1: Detailed experimental procedure to calculate total charge curves.

Voltammetric charges can be obtained from the integration of the voltammogram as indicated in equation (3) of the manuscript. In this equation, $q(E^*)$ represents the integration constant required to extract absolute charge values from the relative values or charge increments resulting from the integration of the voltammogram between two given limits. This value can be obtained from the displaced charge as indicated in equation (2). To avoid the influence of a possible small offset current either due to a miscalibration of the potentiostat or to the effect of traces of oxygen, the average of the positive and negative current is used in the integration of equation (3).

For Pt(111), the displaced charge at 0.10 V stands for 142 µC cm^{-2}, and, according to equation (2), q(0.1 V)=-142 µC cm^{-2}. In combination with the charges obtained from the integration of the voltammogram, the total charge curve shown in red in figure 1 is obtained, resulting in a potential of zero total charge of 0.34 V. In the double layer region, i.e., at 0.51 V, the total charge amounts to 25 µC cm^{-2}. Since no adsorption processes take place in this potential region, this charge value can be attributed to a purely capacitive (ionic) charge. In contrast, the charge at 0.10 V contains two contributions, namely the true free ionic charge and the charge associated with hydrogen adsorption, according to $q=\sigma_M-F\Gamma_H$, where σ_M is the true electronic charge on the metal and Γ_H is the hydrogen

surface excess. According to this relationship, it becomes necessary to differentiate between total charge, q, and free charge, σ_M.

For Pt(311), the displaced charge at 0.10 V and 0.40 V amounts to 90 μC cm^{-2} and -39 μC cm^{-2}, respectively, while the voltammetric charge integrated between 0.10 and 0.45 V amounts to 130 μC cm^{-2}. With these values, it can be verified that, within the experimental errors:

$$\int_{0.1\,\mathrm{V}}^{0.40\,\mathrm{V}} \frac{j}{v} dE = q_{dis}\left(0.1\,\mathrm{V}\right) - q_{dis}\left(0.40\,\mathrm{V}\right)$$

Figure 2 shows the excellent agreement between the CO displacement charges (red dots) and the total charges obtained from the voltammetric integration using the displaced charge at 0.1 V as the integration constant (black curve).

Calculation of theoretical charges for the stepped surfaces.

For the surfaces within the series Pt(S)[n(111)×(100)], whose Miller indexes are Pt(n+1,n-1,n-1) the length of the unit cell projected on the plane of the terrace, as shown in figure S4, is:

$$L = \left(n - \frac{1}{3}\right) d \frac{\sqrt{3}}{2}$$

and the projected area of the unit cell is:

$$S' = \left(n - \frac{1}{3}\right) d^2 \frac{\sqrt{3}}{2}$$

Taking into account that there is only a single step atom in each unit cell, the density of steps per unit area can be calculated as:

$$N = \frac{1}{S'} = \frac{2}{d^2\sqrt{3}} \frac{1}{n - \frac{1}{3}}$$

and the charge corresponding to the exchange of one electron per step site is then:

$$q'^{step}_{(n+1,n-1,n-1)} = \frac{e}{S'} = \frac{2e}{d^2\sqrt{3}} \frac{1}{n - \frac{1}{3}}$$

This charge is projected on the plane of the (111) terrace, which is tilted with respect to the considered surface. The true area, S, and the projected area, S', are related according

to the cosine of the angle α between the surface (n+1,n-1,n-1) and the (111) plane according to:

$$S' = S\cos(\alpha) = S\frac{3n-1}{\sqrt{9n^2-6n+9}}$$

Thus, the charge corresponding to the exchange of one electron per step site (equation (6) of the manuscript) can be written as:

$$q^{step}_{(n+1,n-1,n-1)} = \frac{e}{S} = \frac{2e}{d^2\sqrt{3}}\frac{1}{n-\frac{1}{3}}\cos(\alpha) = \frac{q_{\mathrm{Pt}(111)}}{n-\frac{1}{3}}\cos(\alpha)$$

In this equation, the term $\frac{2e}{d^2\sqrt{3}}$ is the charge associated with the exchange of one electron per unit cell on the (111) surface, $q_{Pt(111)}$.

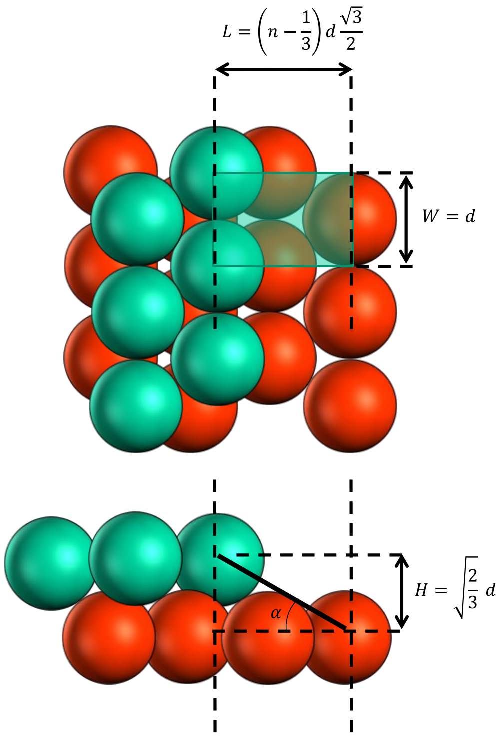

Figure S2. Hard-sphere model of the Pt(n+1,n-1,n-1) surfaces.

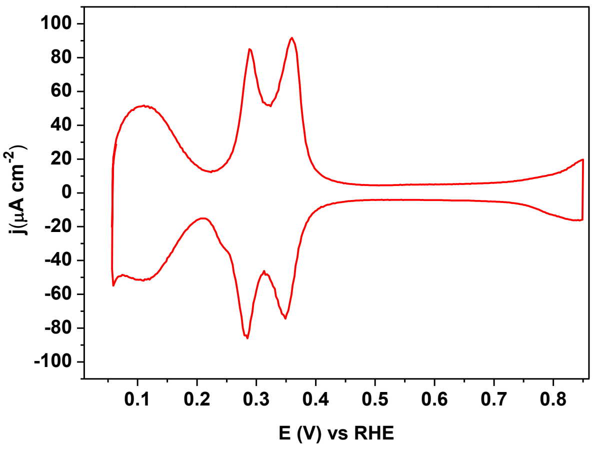

Figure S3. Cyclic voltammetry for Pt(311) in 0.1 M $HClO_4$ solution up to 0.85 V. It is possible to observe that, unlike Pt(111) and long terrace surfaces, Pt(311) shows almost no OH adsorption current contribution states at potential values more positive that the peaks attributed to the potential for the (100) steps.

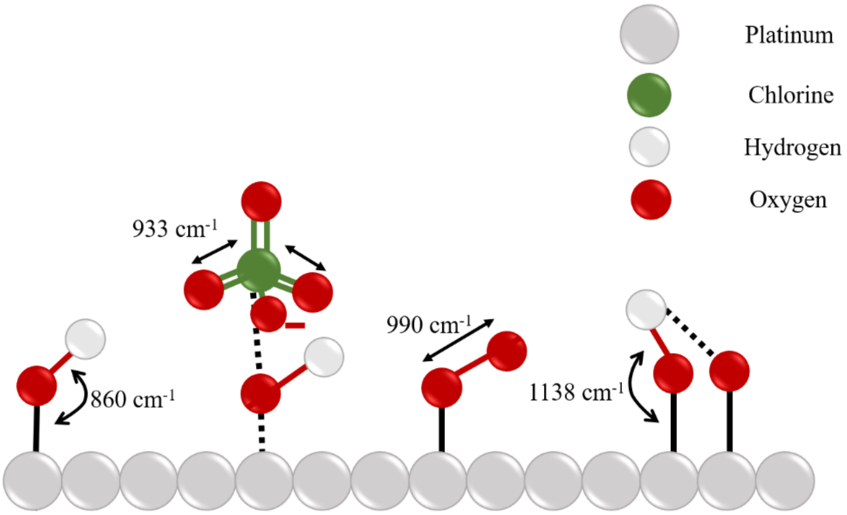

Figure S4. Schematic representation of species adsorped at potentials above 0.25 V based on *in situ* SHINERS results.

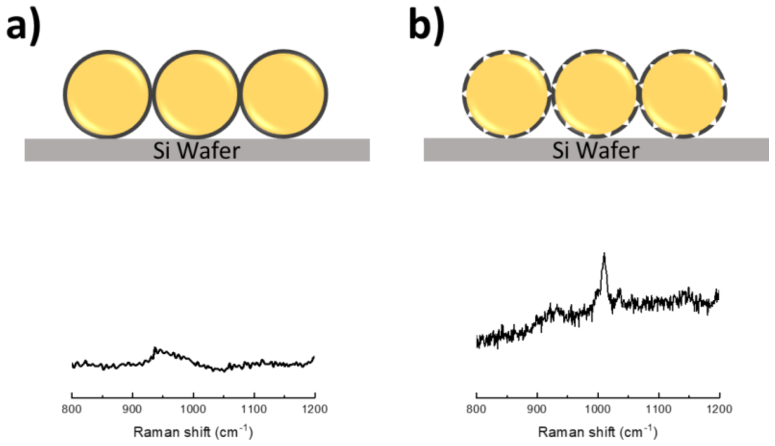

Figure S5. Pinhole test performed for shell isolated nanoparticles (SHINs). SHINs used throughout the experiment were tested for pinholes by depositing the nanoparticles onto a Si surface and measuring the Raman response of pyridine. a) SHINs with no pinholes showed no pyridine characteristic bands, whereas b) two characteristic bands at 1011 and 1036 cm^{-1} corresponding to the ring breathing mode and asymmetric stretch, respectively[1,2] of pyridine adsorbed onto Au were observed with the presence of pinholes. Consequently, these SHINs were discarded and only SHINs with no pinholes were used for the study presented in this paper.

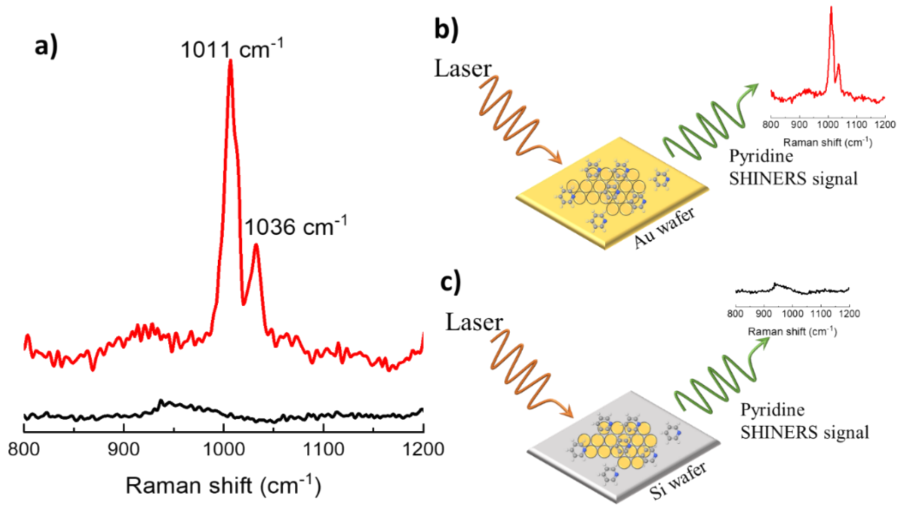

Figure S6. Pinhole and enhancement tests were performed before and after every measurement to ensure an adequate performance of the SHINs used. No pinholes were observed for SHINs deposited on Si wafer while a large enhancement is observed for SHINs deposited onto Au wafer for pyridine as a probe molecule.

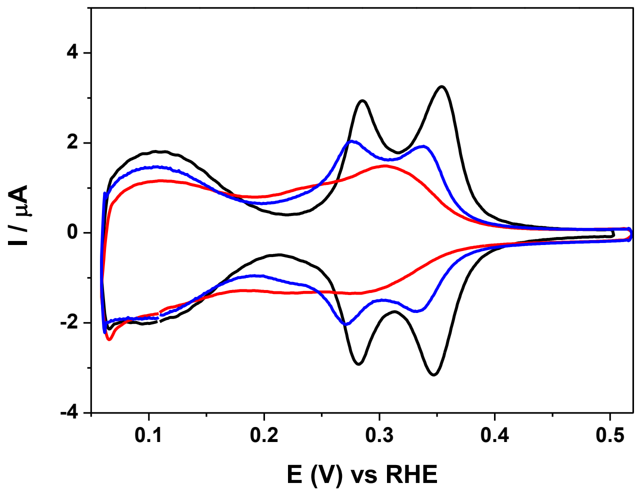

Figure S7. Characterization of Pt(311) in 0.1 M $HClO_4$ without (black line) and with deposited SiO_2-coated SHINs onto its surface before (red line) and after (blue line) 5 minutes of hydrogen evolution reaction treatment.

References

1. Galloway, T. A., Cabo-Fernandez, L., Aldous, I. M., Braga, F. & Hardwick, L. J. Shell isolated nanoparticles for enhanced Raman spectroscopy studies in lithium-oxygen cells. *Faraday Discuss.* **205**, 469–490 (2017).

2. Tian, X. D., Liu, B. J., Li, J. F., Yang, Z. L., Ren, B. & Tian, Z. Q. SHINERS and plasmonic properties of Au Core SiO_2 shell nanoparticles with optimal core size and shell thickness. *J. Raman Spectrosc.* **44**, 994–998 (2013).
